# Coronavirus disease 2019-related Kawasaki syndrome: a case report

**DOI:** 10.1186/s13256-022-03589-z

**Published:** 2022-11-09

**Authors:** Mohammad Hasan Aelami, Abdolreza Malek, Amin Saeidinia

**Affiliations:** 1 Department of Pediatrics, Faculty of Medicine, Mashhad University of Medical Sciences, Mashhad, Iran; 2 Pharmaceutical Research Center, Fakouri Boulevard, Booali Research Center, Mashhad University of Medical Sciences, Mashhad, Iran

**Keywords:** COVID-19, Kawasaki, Multisystem inflammatory syndrome in children (MIS-C)

## Abstract

**Background:**

Coronavirus disease 2019 has changed the pattern of some diseases in the world, especially in pediatrics. Despite data suggesting that the pediatric population is less affected by coronavirus disease-19 infection, new concerns have been raised owing to reported cases with hyperinflammatory conditions such as Kawasaki disease.

**Case presentation:**

We report herein the case of a pediatric patient diagnosed and treated for classic Kawasaki disease in the setting of confirmed coronavirus disease 2019 infection. She was an 8-year-old, previously healthy, and fully immunized Iranian girl who initially presented to the pediatric emergency department with 5 days of intermittent fever, followed by abdominal pain, nausea, and vomiting. She was admitted for fever and abdominal pain to the surgery service of Akbar Hospital with suspected appendicitis.

**Conclusions:**

This case report may serve as a useful reference to other clinicians caring for pediatric patients affected by coronavirus disease 2019 infection. Standard therapeutic interventions for Kawasaki disease must be performed to prevent critical coronary aneurysm-related complications in the coronavirus disease 2019 era.

## Background

The rapid spread of coronavirus disease 2019 (COVID-19) caused by severe acute respiratory syndrome coronavirus 2 led to a global pandemic, with infected individuals of all ages residing in almost every country in the world. The pediatric population appears to be affected in much smaller proportions than adults, with only 2% of cases described in those younger than 20 years [1–3]. In an epidemiological report, authors described 731 confirmed COVID-19 cases in the pediatric population, with more than 90% characterized as asymptomatic, mild, or moderate infection [4]. They looked at a total of 2143 patients, 1412 of whom had suspected but unconfirmed COVID-19 infection, but there was little description of coincidence with other clinical conditions and very limited cases reported of concurrent Kawasaki disease (KD). Despite data suggesting that the pediatric population is less affected by COVID-19, new concerns have been raised owing to reported children with hyperinflammatory conditions such as KD, toxic shock syndrome (TSS), and macrophage activation syndrome (MAS)/hemophagocytic lymphohistiocytosis (HLH) [5]. MIS-C and KD display several notable epidemiological differences. The median age of children with MIS-C is 8–9 years, whereas most children with KD are below 5 years with a median age of 3 years and a peak of incidence in infants less than 1 year old. Children who develop MIS-C are generally older than those with severe pediatric COVID-19 infection, although the lower mean age of patients with severe pediatric COVID-19 is largely due to the high proportion of infants among them. In both MIS-C and severe pediatric COVID-19 infection, most patients were previously healthy, although a possible association with obesity in adolescents has been suggested. KD affects boys more than girls, whereas no clear sex predominance has been observed in MIS-C patients. The fatality rate of MIS-C has been estimated at about 1–2%, much higher than that reported for KD [6].

We describe herein the case of a pediatric patient diagnosed and treated for classic KD in the setting of confirmed COVID-19 infection.

## Case presentation

**Fig. 1:**
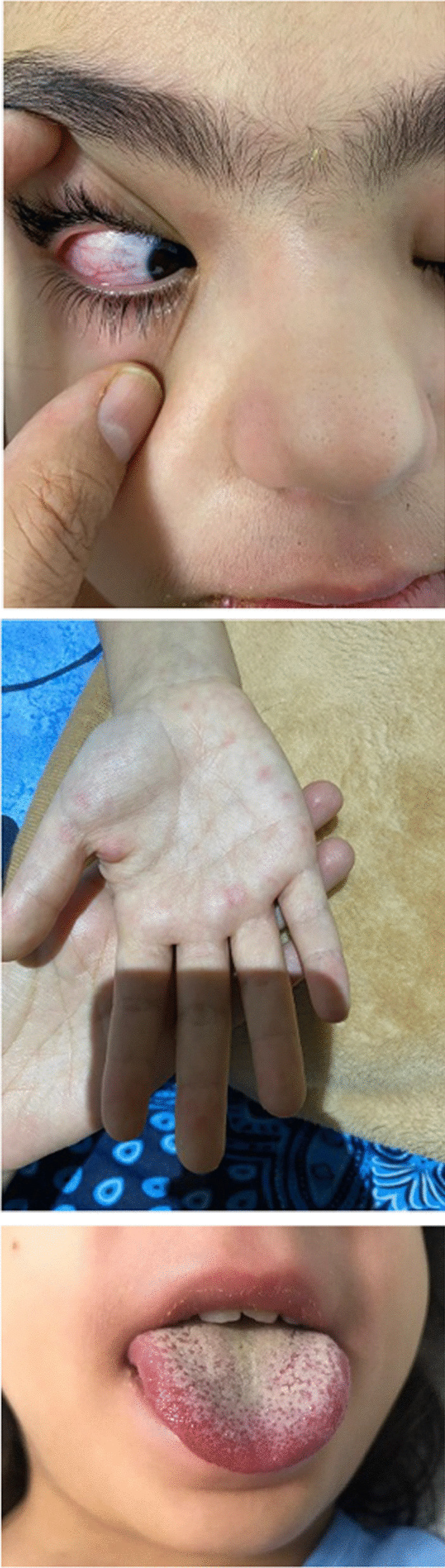
Nonpurulent conjunctivitis, Strawberry tongue, palmar rash in the case of Kawasaki disease

The patient was an 8-year-old, previously healthy, and fully immunized Iranian girl who was initially presented to the pediatric emergency department with 5 days of intermittent fever, followed by abdominal pain, nausea, and vomiting. She had not attended school within the last 6 months and was cared for by her parents. She was an active child, but staying home most of the time because of the COVID-19 pandemic. Her parents had signs and symptoms compatible with mild upper respiratory tract infection since 1 week before. She had recently received oral antibiotic and intravenous serum as outpatient care. She was admitted with fever and abdominal pain in the surgery service of Akbar Hospital with suspected appendicitis. Nausea and vomiting persisted. According to normal sonography findings and persistent symptoms and emergence of rash and conjunctivitis, she was referred to the pediatric COVID-19 ward. She did not have cough, congestion, or rhinorrhea. After the third day, she received co-trimoxazole and ondansetron. Physical examination revealed bilateral nonpurulent conjunctivitis, erythematous throat, palmar erythema, strawberry tongue, and erythema multiform rash (Fig. 1) without hypotension. There was no lymphadenopathy or retraction breathing, with normal respiratory sounds. There were no focal signs of infection. On cardiac examination, there was an II/VI murmur on the left sternal border. According to fever (38.6 °C) and pandemic state of COVID-19, she underwent lung high-resolution computed tomography (HRCT), which was not indicative for COVID-19 infection. Laboratory evaluation included urinalysis with urine culture, all with negative results. On laboratory examination, ESR was 67 mm/h, CRP 166 mg/L, platelets 192 × 10^9^/L, hemoglobin 10.2 g/dL and WBC 10.3×10^9^/L (PMN: 82%). Moreover, other routine biochemistry and liver function tests yielded normal findings.

She received IVIG (2 g/kg; 1 g/kg on two consecutive days) and high-dose acetylsalicylic acid (20 mg/kg four times daily) according to rheumatology consultation. Echocardiography showed no pathology related to coronary arteries. Besides, respiratory tract viral infections were evaluated by reverse transcription polymerase chain reaction (RT-PCR) test for COVID-19 and blood culture, all with negative results. There was no history of recent travel. Nonetheless, her parents had flu-like symptoms along with hypogeusia and anosmia. She was hospitalized for 5 days. The day before discharge, serologic tests for COVID-19 infection in her parents (serum IgM more than 1.1) and the child herself became positive (serum IgM COVID-19 of 2.1). Consequently, the public health department was notified, and the family was instructed to quarantine at home for 14 days from the positive test result date. She was discharged on low-dose acetylsalicylic acid (3 mg/kg daily), with plans to follow-up with pediatric cardiology for repeat echocardiographic evaluation 2 weeks later. On follow-up echocardiography, there was no aortic artery involvement.

## Discussion

To the best of the authors’ knowledge, this is the first report of Kawasaki disease in the setting of COVID-19 infection in Iran. Kawasaki disease is an acute vasculitis in childhood and the major etiology of acquired pediatric cardiac disease in developed countries. In total, 50% of patients are diagnosed during childhood younger than 2 years and 80% below 5 years [7]. The diagnosis of “classic” Kawasaki disease is made in patients presented with fever for at least 5 days together with minimally four of five clinical criteria in the absence of an alternate diagnosis [8]. Despite a mass of research, the etiology of Kawasaki disease remains unclear. There are some hypotheses on its etiology; some speculate that infectious agents might trigger the disease, while others believe that winter or spring seasons are its initiators [7]. There are some implications about the association between viral respiratory infections and Kawasaki disease.

In different previous studies, respiratory viral infection test was positive in 9–42% of patients with Kawasaki disease [9–11]. In some clinical aspects, multisystem inflammatory syndrome in children (MIS-C) and KD have the same presentations. In both of them, fever, rash, oropharynx congestion, and red eyes have been reported. We still do not know the extent of overlap between KD and MIS-C. Some differences are age distribution, cardiac involvement, inflammatory markers, and ethnic variations [12]. MIS-C is a rare but severe complication of COVID-19, which can make quick clinical regression in patients. Pediatric patients with persistent fever and positive familial history of COVID-19 are at higher risk of MIS-C. It was demonstrated that lower level of serum albumin and older age are the two independent predictors of children who need pediatric intensive care [13].

Because of the course of disease after admission, history of COVID-19 infection in parents, and the pandemic status, Kawasaki was thought to be triggered by the COVID-19 virus. Although the number of reported cases is growing, there are many knowledge gaps regarding various aspects of COVID-19 infection such as its epidemiology and clinical characteristics. The face of the disease is changing fast, particularly in the pediatric population. To date, the most common pediatric presentation of COVID-19 is an array of signs and symptoms including completely asymptomatic to symptoms of acute upper respiratory tract infection such as fever, fatigue, cough, sore throat, rhinorrhea and congestion, and shortness of breath. In more severe cases, gastrointestinal symptoms might develop and patients can progress to respiratory failure, shock, coagulation dysfunction, and renal injury [4]. In some COVID-19 cases, abdominal pain might mislead surgeons to appendicitis or other differential diagnoses of acute abdomen [14]. In this case, we excluded appendicitis by ultrasonography and persistence of rash and conjunctivitis. Fourkiki *et al.* reported a cohort of six Swiss children with SARS-CoV-2-related MIS-C who presented with clinical features compatible with incomplete KD and TSS associated with a cytokine storm. They demonstrated higher IL1RA levels in five of the six patients, whereas IL-6 serum levels were increased in only three of six patients. All patients received high-dose IVIG, except one who received anakinra. In addition to all these antiinflammatory medications, two patients received one dose of anti-IL6. They suggested the use of anakinra as an alternative to steroids in these children, because of the high IL-1RA levels [15]. In another study, Acka *et al.* evaluated children with typical and atypical KD likely associated with COVID-19 infection. They reported four children with Kawasaki-like disease, probably associated with COVID-19. The clinical features were consistent with incomplete KD in three patients. SARS-CoV-2 RT-PCR was positive in one, and the serology was positive in another with negative RT-PCR finding. They used corticosteroids, anakinra, IVIG, and acetylsalicylic acid. Three patients recovered after treatment, while one died [16]. Despite the fact that, in a previous investigation of 320 children with Kawasaki-like disease associated with COVID-19, SARS-CoV-2 RT-PCR result was negative in 65.5%, the serology finding was positive in 83.8% [16].

With the spread of COVID-19 infection worldwide, clinical criteria for ×COVID-19 only may be restricted to those with respiratory symptoms, because of some constraints such as testing availability or financial issues. In pediatrics, with the clinical spectrum yet to be clearly defined, patients presenting with fever alone or primarily with other organ system involvement such as gastrointestinal symptoms may be missed if testing is restricted to those with respiratory complaints. Another important point in the COVID-19 era is that children below 12 years with multi-inflammatory syndrome related to COVID-19 (MIS-C) are more likely to present with Kawasaki disease-like symptoms such as conjunctival injection, rash, and oral mucosal changes than adolescents with MIS-C, which may need ICU admission [17]. Therefore, close evaluation of such patients is necessary.

## Conclusion

This case report may serve as a useful reference for other clinicians caring for children affected by COVID-19 as understanding of its clinical presentations continues to evolve. Further description of the clinical course of pediatric patients diagnosed with COVID-19 remains necessary, particularly regarding the potential association with KD. Standard therapeutic interventions for KD must be performed to prevent critical coronary aneurysm-related outcomes owing to delayed diagnosis in the COVID-19 era.

## Data Availability

Not applicable.

## References

[1] Wu Z, McGoogan JM (2020). Characteristics of and important lessons from the coronavirus disease 2019 (COVID-19) outbreak in China: summary of a report of 72 314 cases from the Chinese Center for Disease Control and Prevention. JAMA.

[2] Keihanian F, Poorzand H, Saeidinia A, Eshraghi A (2020). Echocardiographic and electrocardiographic findings in COVID-19 patients: a cross-sectional study. Int J Cardiovasc Imaging.

[3] Ahanchian H (2021). Death due to COVID-19 in an infant with combined immunodeficiencies. Endocr Metab Immune Disord Drug Targets.

[4] Dong Y (2020). Epidemiological characteristics of 2143 pediatric patients with 2019 coronavirus disease in China. Pediatrics.

[5] Haslak F (2021). A recently explored aspect of the iceberg named COVID-19: multisystem inflammatory syndrome in children (MIS-C). Turk Arch Pediatrics.

[6] Sancho-Shimizu V (2021). SARS-CoV-2-related MIS-C: a key to the viral and genetic causes of Kawasaki disease?. J Exp Med.

[7] Rowley AH, Shulman ST (2018). The epidemiology and pathogenesis of Kawasaki disease. Front Pediatr.

[8] Kumrah R (2020). Immunogenetics of Kawasaki disease. Clinic Rev Allerg Immunol.

[9] Turnier JL (2015). Concurrent respiratory viruses and Kawasaki disease. Pediatrics.

[10] Kim JH (2012). Detection rate and clinical impact of respiratory viruses in children with Kawasaki disease. Korean J Pediatr.

[11] Jordan-Villegas A (2010). Concomitant respiratory viral infections in children with Kawasaki disease. Pediatr Infect Dis J.

[12] Lee M-S (2021). Similarities and differences between COVID-19-related multisystem inflammatory syndrome in children and kawasaki disease. Front Pediatr.

[13] Haslak F (2021). Clinical features and outcomes of 76 patients with COVID-19-related multi-system inflammatory syndrome in children. Clin Rheumatol.

[14] Khakshour A, Saeidinia A, Ghanbari G (2021). Atypical presentation of COVID-19 infection as acute abdomen in children: a case series. Clin Case Rep.

[15] Fouriki A (2021). Case report: case series of children with multisystem inflammatory syndrome following SARS-CoV-2 infection in Switzerland. Front Pediatr.

[16] Akca UK (2020). Kawasaki-like disease in children with COVID-19. Rheumatol Int.

[17] Dufort EM (2020). Multisystem inflammatory syndrome in children in New York State. N Engl J Med.

